# The expression profile of virus-recognizing toll-like receptors in natural killer cells of Cypriot multiple sclerosis patients

**DOI:** 10.1186/s13104-020-05300-1

**Published:** 2020-09-29

**Authors:** Elie Deeba, Anastasia Lambrianides, Marios Pantzaris, George Krashias, Christina Christodoulou

**Affiliations:** 1 Cyprus School of Molecular Medicine, The Cyprus Institute of Neurology and Genetics, 6 International Airport Avenue, 2370 Nicosia, Cyprus; 2 Neurology Clinic C, The Cyprus Institute of Neurology and Genetics, Nicosia, Cyprus; 3 Department of Molecular Virology, The Cyprus Institute of Neurology and Genetics, P.O.Box 23462, 1683 Nicosia, Cyprus

**Keywords:** Multiple sclerosis, Toll-like receptor, Flow cytometry, Natural killer cells

## Abstract

**Objective:**

The exact aetiology of multiple sclerosis (MS) remains elusive, although several environmental and genetic risk factors have been implicated to varying degrees. Among the environmental risk factors, viral infections have been suggested as strong candidates contributing to MS pathology/progression. Viral recognition and control are largely tasked to the NK cells via TLR recognition and various cytotoxic and immunoregulatory functions. Additionally, the complex roles of different TLRs in MS pathology are highlighted in multiple, often contradictory, studies. The present work aims to analyse the TLR expression profile of NK cells isolated from MS patients. Highly purified CD56^+^CD3^−^ NK cells isolated from peripheral blood of MS patients (n = 19) and healthy controls (n = 20) were analysed via flow cytometry for their expression of viral antigen-recognizing TLRs (TLR2, TLR3, TLR7, and TLR9).

**Results:**

No difference was noted in TLR expression between MS patients and healthy controls. These results aim to supplement previous findings which study expressional or functional differences in TLRs present in various subsets of the immune system in MS, thus aiding in a better understanding of MS as a complex multifaceted disease.

## Introduction

There is wide consensus defining multiple sclerosis (MS) as a chronic demyelinating disease, with studies showing apparent aspects of autoimmunity [1, 2]. The risk factors that play a role in disease pathogenesis vary widely, whether genetic or environmental risk factors, working either separately or in combinations; however, the exact mechanisms of how these might interact remain unknown [3–6]. Some of the environmental risk factors include age, gender, geographical location, and diet and lifestyle [4–6]. Viral infections have taken centre stage in recent years as one of the major environmental risk factors implicated in MS [4, 7, 8]. Examples include the Epstein-Barr virus (EBV), human cytomegalovirus (HCMV), varicella zoster virus (VZV), human herpes virus-6 (HHV-6), and even human endogenous retroviruses (HERV) [9].

Natural killer (NK) cells are classified as group I innate lymphoid immune cells [10] that have both cytotoxic and immunoregulatory functions depending on their subsets [11, 12]. NK cells have emerged in research in the past two decades as a possible player in the pathology of MS. One study showed the exacerbation of experimental autoimmune encephalitis (EAE) as a result of the depletion of NK cells [13, 14]. Such observations have also been noticed in MS patients to a certain degree due, in part, to the wide variability in criteria and protocols used to classify NK cell activity and frequency in patients, as well as variability in patient selection [13, 15]. A recent study found rapid reconstitution of NK cells following autologous hematopoietic stem cell transplantation in relapsing remitting MS (RRMS), which curbed an overexpansion of the effector memory T cell subset, Th17 cells [16]. Many of these findings have to be further investigated due to the complexity of both NK cell subsets and functions as well as complexity of MS as a whole [15, 17, 18].

NK cells play a key role in host defence against viral infections, including those arising from members of the herpesvirus family [19–21]. The ability of NK cells to respond to viral stimuli relies on a series of germ-line encoded receptors, among them the toll-like receptors (TLRs), which can be expressed on the cell surface or within intracellular compartments [22]. TLRs that are known to recognize viral antigens include TLR2, TLR3, TLR7, and TLR9 [23].

It is safe to hypothesize that the lack, or even dysregulation, of any one of the TLRs could have severe repercussions on the ability of the immune cells, including NK cells, to control infections, or may possibly aid in the pathogenesis of diseases such as MS. Given the importance of NK cells in viral control and its suggested association with MS, we aimed to evaluate, for the first time, the expressions of TLR2, TLR3, TLR7 and TLR9 in the NK cells of Cypriot MS patients.

## Main text

### Study population

The study consisted of 19 patients with clinically definite MS and 20 healthy controls (HCs), who were matched for age and gender. Blood samples were collected from MS patients during their routine follow-up visits at the neurology clinic C of The Cyprus Institute of Neurology and Genetics. As described previously [24], the inclusion criteria were: (1) individuals above 18 years of age; (2) MS patients with clinically definite multiple sclerosis (CDMS) and clear relapsing–remitting clinical course; (3) patients not experiencing any relapse symptoms at the time of blood collection; (4) availability of a detailed clinical history (age of onset, disease duration, Expanded Disability Status Scale (EDSS) score, and treatments received); (5) being born and having resided in Cyprus from birth to early adult life at the least. Exclusion criteria were: (1) patients having suffered a relapse episode within 30 days before enrolment and/or blood collection; (2) inability or unwillingness to provide informed consent; (3) a history of alcohol or drug abuse; (4) pregnancy. The demographic details and clinical characteristics (EDSS, diseases duration, treatment at time of blood collection) of the MS patients and HCs can be found in Additional file 1: Table S1.

### NK staining and evaluation via flow cytometry

Ethylene diamine tetraacetic acid (EDTA)-anticoagulated venous peripheral blood was collected and peripheral blood mononuclear cells (PBMCs) were extracted by Lymphoprep (Accu-Prep, 1.077 g/mL, Accurate Chemical and Scientific Corp., USA) gradient centrifugation, following the manufacturer’s instructions.

In a v-bottomed 96 well plate, 1 × 10^6^ PBMCs per well were resuspended in 100 µL of cell staining buffer (Biolegend, Germany), and incubated first for 10 min on ice with human FcR blocking reagent (Miltenyi Biotec, Germany), followed by 1 h at 4 °C with antibodies against CD3 (FITC, clone HIT3a, Biolegend, Germany) and CD56 (PE/Cy5, clone MEM-188, Biolegend, Germany). The excess antibodies were then washed off, and the cells were fixed with 2% paraformaldehyde (PFA) (Sigma-Aldrich, Germany) in 1× PBS for 20 min at room temperature. PFA was then washed off, and the cells were permeabilized using intracellular staining perm wash buffer (Biolegend, Germany), following the manufacturer’s instructions. For intracellular staining, the cells were then resuspended in 100 µL of the perm wash buffer and incubated separately for 1 h at room temperature with antibodies against TLR2 (PE, clone 2B4A1, Invitrogen, USA), TLR3 (PE, clone TLR3.7, Invitrogen, USA), TLR7 (PE, clone 4G6, Invitrogen, USA), TLR9 (PE, clone eB72-1665, Invitrogen, USA). Excess antibodies were then washed off and the cells were resuspended in 1× PBS for flow cytometric analysis.

**Fig. 1:**
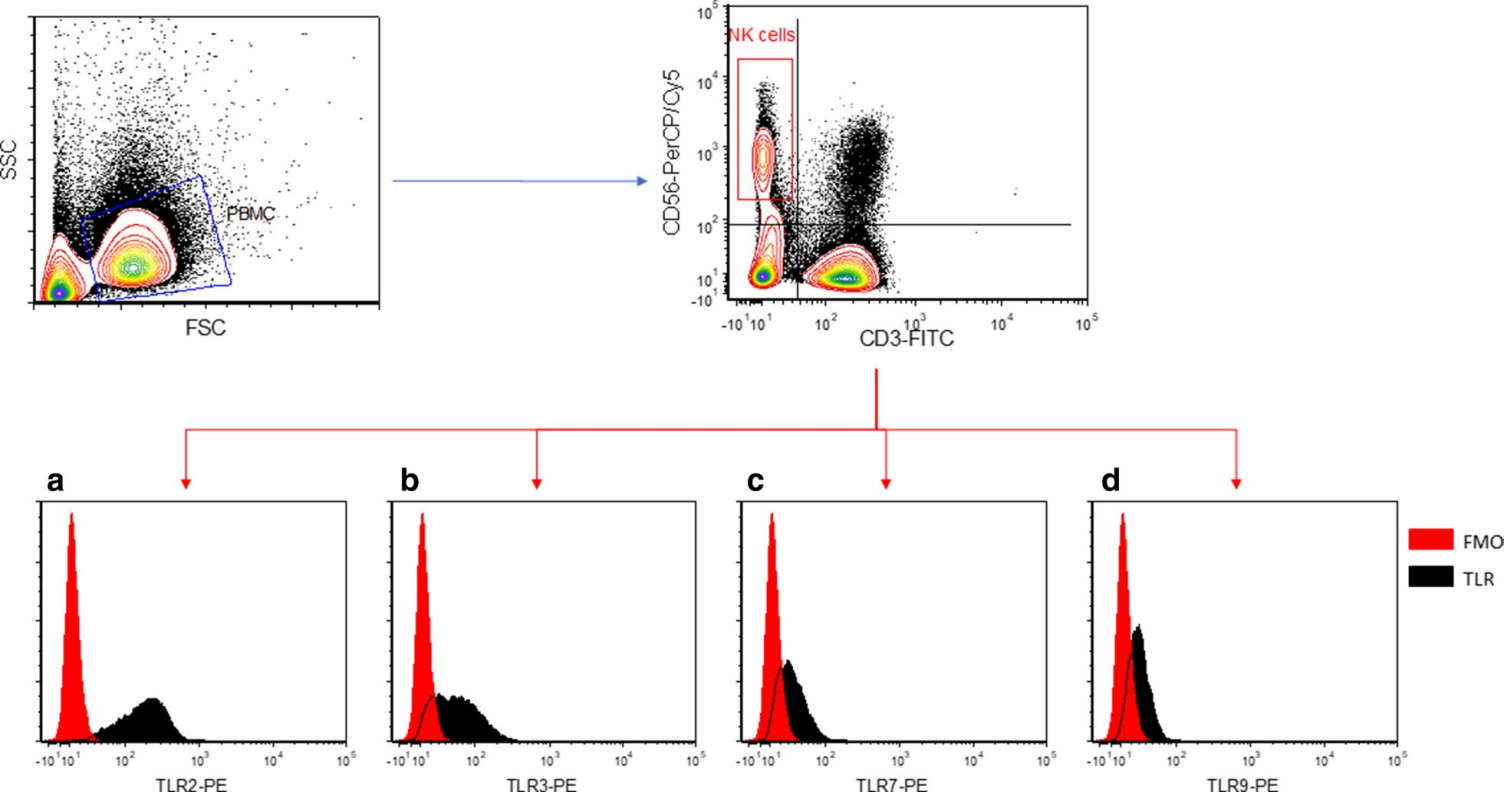
Representative workflow contour plots and histograms from flow cytometry measurements of TLR2, TLR3, TLR7 and TLR9 expressions in NK cells. The blue gate in the FSC/SSC plot represents the PBMC population, and the red gate in the CD3-FITC/CD56-PerCP/Cy5 plot represents the CD56^+^ CD3^−^ NK cells. The red histogram represents background fluorescence from the fluorescence-minus-one (FMO) sample, and the black histogram represents the fluorescence from the TLR2-PE (**a**), TLR3-PE (**b**), TLR7-PE (**c**), and TLR9-PE (**d**) stainings

Flow cytometry was performed using a CyFlow cube 8 (Sysmex-Partec, Germany). The PBMC population was gated based on the FSC/SSC properties (Fig. 1), and 100,000 events were acquired for analysis. The experimental setup included a fluorescence-minus-one (FMO) sample, i.e. cells stained with anti-CD3 and anti-CD56 only, to be used as the control overlay for TLR expression analysis, as well as single stained controls to be used for post-acquisition computed compensation. Data analysis was performed using FCS express 4, Research edition (De Novo software, CA, USA). The CD56^+^CD3^−^ population was identified as NK cells (Fig. 1), and TLR expression of this population was measured via 2 parameters: (a) the percentage of cells which are positive with respect to the FMO overlay (%positivity) using the software-calculated algorithm, and (b) the percentage mean fluorescence intensity difference compared with the FMO overlay (%MFI) using the formula $\frac{{{\text{MFI of TLR stained sample }} - {\text{ MFI of FMO sample}}}}{{\text{MFI of FMO sample}}} \times 100$.

### Statistical analysis

The Mann–Whitney U test was used for age matching, and the Fisher’s exact test was used for gender matching. The Mann–Whitney U test was also used to assess significance (*p* < 0.05) in TLR expression differences between the studied groups in terms of both %positive and %MFI parameters.

### Results

**Fig. 2:**
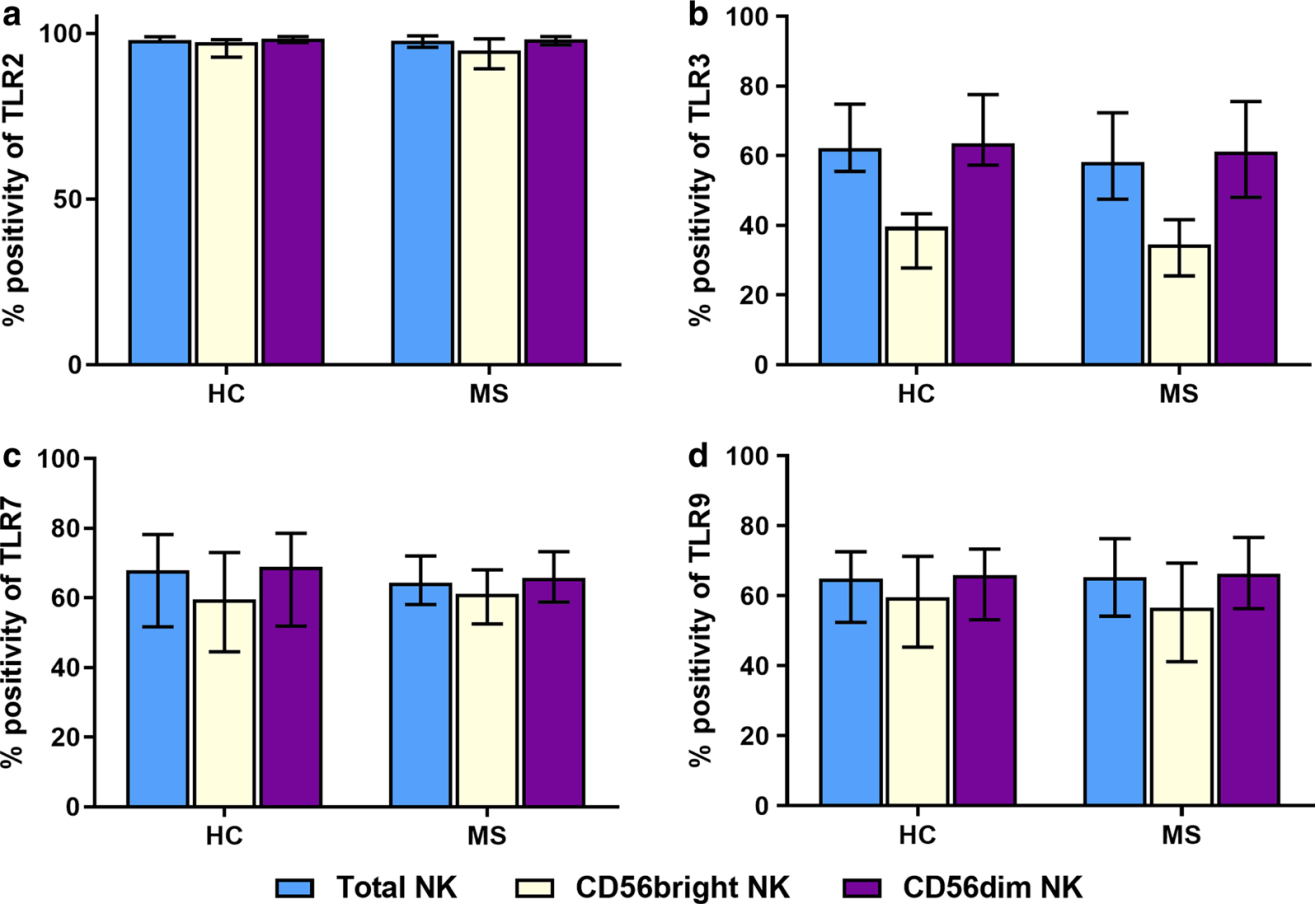
The percentage positivity distributions of TLR2 (**a**), TLR3 (**b**), TLR7 (**c**), and TLR9 (**d**) in MS patients (n = 19) versus healthy controls (HCs) (n = 20). The percentage positivity is compared in total NK cells, the CD56^bright^ NK subpopulation, and the CD56^dim^ NK subpopulation. Bars represent the median and the interquartile range for each group

**Fig. 3:**
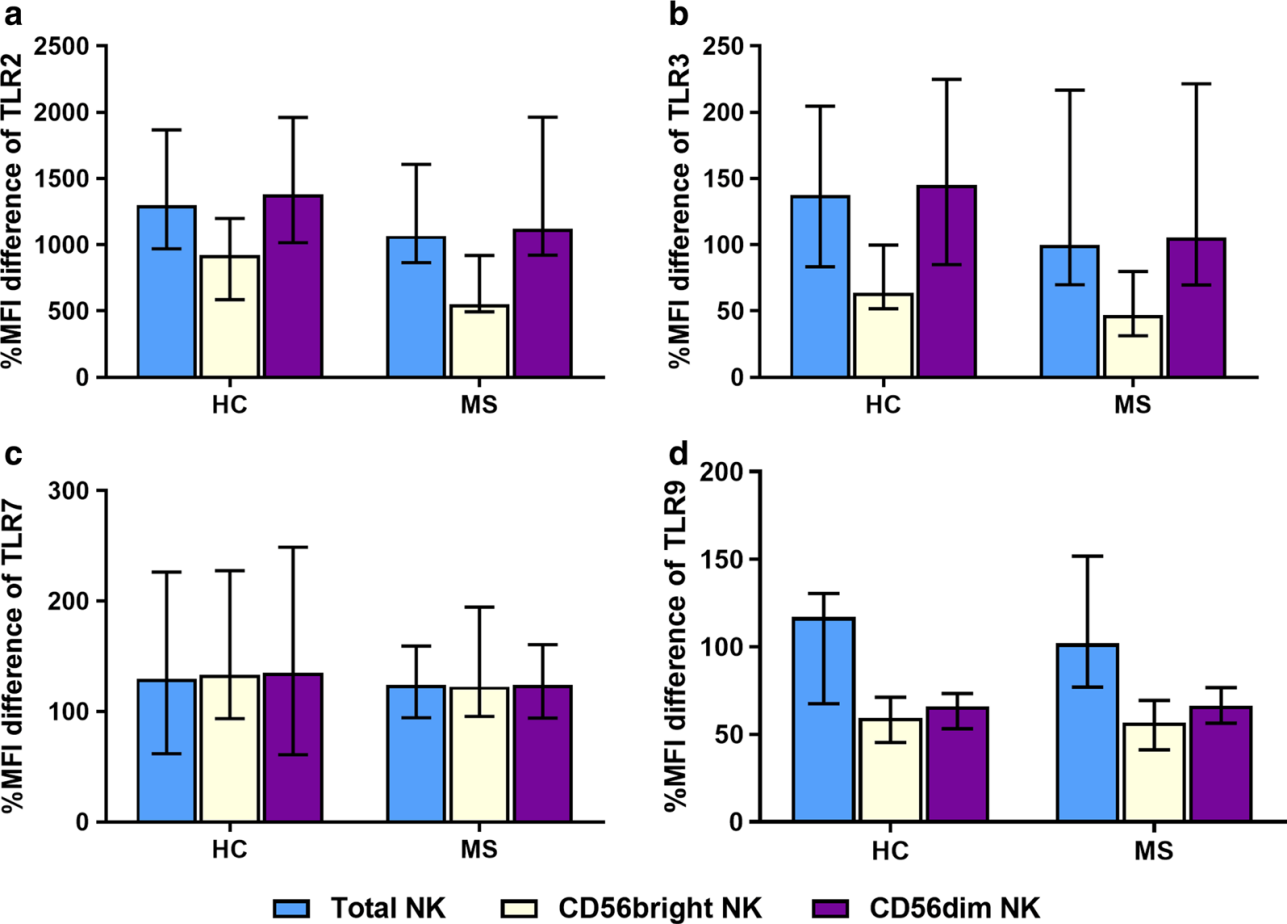
The percentage mean fluorescence intensity (MFI) difference distributions of TLR2 (**a**), TLR3 (**b**), TLR7 (**c**), and TLR9 (**d**) in MS patients (n = 19) versus healthy controls (HCs) (n = 20). The percentage MFI difference is compared in total NK cells, the CD56^bright^ NK subpopulation, and the CD56^dim^ NK subpopulation. Bars represent the median and the interquartile range for each group

The percentage expressions of TLR2, 3, 7, and 9 in the total NK populations represented by the percentage positivity (%positivity) were not significantly different among MS and HCs samples (Fig. 2). Similarly, the expressions of the TLRs per NK cell represented by the percentage MFI difference (%MFI) were also not significantly different among MS and HCs samples (Fig. 3).

Further gating that discerns the CD56^bright^ and the CD56^dim^ populations of the NK cells was performed. Upon analysing the %positivity (Fig. 2) and %MFI (Fig. 3) of the two separate subpopulations, no significant differences were found among MS and HC samples.

On another note, the MS group was separated into MS patients receiving medication versus MS patients not receiving any medication at the time of sampling. Comparing %positivity and %MFI between these 2 groups showed no significant difference in TLR expression in either total NK cells, or the subsets of NK cells (CD56^bright^ and CD56^dim^).

### Discussion

Recent emphasis is being directed towards the relevance of the innate immune system in MS pathogenesis/progression due to the importance of the interplay between the innate and adaptive immunities [25, 26]. Furthermore, specific attention is being given to the effect or role of TLRs in MS [27, 28]. A lack or dysregulation of any one of the TLRs could theoretically have severe repercussions on the ability of the immune cells, including NK cells, to control infections, or may possibly aid in the pathogenesis of diseases such as MS. In concordance with that hypothesis, and taking into consideration the association of viral infections with MS, this study aimed to analyse NK cell expression of viral antigen-recognizing TLRs in MS patients for the first time.

Our results showed that viral antigen-recognizing TLR expression profile of NK cells in MS patients was similar to that of the healthy controls, in terms of percentage of NK cells expressing the TLRs as well as expression per NK cell, regardless of their phenotypic differences (CD56^bright^ or CD56^dim^). Given the specific setup and conditions followed in our methodology, we were not able to validate our initial hypothesis about the possible involvement of specific TLRs in MS. Nevertheless, there are other TLRs as well as other pattern recognition receptors that are expressed and play a role in NK cell immunity [29, 30]. It is essential, therefore, to dissect the different expressional and functional profiles of the immune system and present the findings as to build a better understanding of the different complex pathways implicated in MS pathogenesis/progression.

In fact, studies have focused on specific TLRs in different cell subsets of the immune system in association with MS [31–37]. For instance, Nyirenda et al. found that TLR2 expression is higher in Treg cells of MS patients compared to HCs [38]. Upon stimulation using a TLR2 agonist, reduction of Treg function and Th17-like phenotype skewing occurred in MS patients more than in HCs [38]. Enhanced TLR2 responsiveness to its agonist was reported in monocytes and PBMCs of MS patients [31]. The same study found no differences in TLR2 expression in monocytes of MS patients compared to HCs [31]. In the murine model of MS, experimental autoimmune encephalomyelitis (EAE), the lack of TLR2 in CD4^+^ T cells was shown to ameliorate EAE [32], while inducing TLR2 tolerance via low-levels of a microbiome-derived TLR2 agonist resulted in amelioration of EAE [39]. One study showed an enhanced expression of TLR3 in inflamed CNS tissues [40]. Genetic correlation studies on different TLR3 variants have found no association between the variants and MS [41, 42]. However, we have recently found such an association, i.e. between a TLR3 variant (rs3775291) and MS, in the Cypriot MS population [24]. This discrepancy can be explained by the imbalance in genetic studies that favour North American and North European studies, as opposed to a more diversified approach. Due to the importance of IFN-β in MS [43], TLRs that regulate IFN-β expression play a pivotal role in the development of the disease, as seen by data from EAE models [44]. The TLRs shown to be involved in IFN-β production, include TLR3, 7, and 9 [45]. Additional evidence shows the correlation of TLR9 expression in glial cells with disease severity in EAE [33]. Concurrently in MS patients, a study on TLR7 showed the importance of TLR7 activation via its agonist, alongside administration of exogenous IFN-β, as a means to re-establish proper B cell immunoregulatory signalling in RRMS patients [46]. The study also found a decreased TLR7 gene expression in B cells of RRMS patients which lead to a lowered endogenous IFN-β production by the B cells [46]. Similarly to TLR7, TLR9 was found to have reduced expression in B cells of MS patients, which lead to decreased production of IL-10 by the B cells [47].

At the very least, the results show that TLR expression in NK cells is not affected in Cypriot MS patients. However, considering the many efforts to study TLR expression as well as function in different immune cells separately, future studies may need to focus on whether NK cells respond differently to activation via TLRs in MS, or whether various treatments in MS affect that response. Considering the fact that NK activation by TLR is also dependent on co-stimulatory signals by local cytokines [48], future studies may focus on TLR expression during different disease states and/or in the presence of different co-stimulatory signals, such as IL-2, IL-12, or IFNγ. Studies may also look into the downstream implications of such a response on other immune cells and/or the demyelination and remyelination mechanisms.

## Limitations

- Limited sample pool size.
- Rudimentary classification of NK cell population; other markers could be used to further divide the NK population into more specific subpopulations.

## Supplementary information

**Additional file 1: Table S1.** Demographic and clinical characteristics of MS patients and healthy controls. The Mann-Whitney U test was used for age matching, and the Fisher’s exact test was used for gender matching.

## Data Availability

The datasets used and/or analysed during the current study are available from the corresponding author on reasonable request.

## Abbreviations

%MFI: Percentage mean fluorescence intensity
%positivity: Percentage positivity
CDMS: Clinically definite multiple sclerosis
CNS: Central nervous system
EAE: Experimental autoimmune encephalitis
EBV: Epstein-Barr virus
EDSS: Expanded disability status scale
EDTA: Ethylene diamine tetraacetic acid
FMO: Fluorescence minus one
FSC: Forward scatter
HC: Healthy controls
HCMV: Human cytomegalovirus
HERV: Human endogenous retrovirus
HHV-6: Human herpesvirus 6
IFN-β: Interferon- β
MBP: Myelin basic protein
MS: Multiple sclerosis
PBMC: Peripheral blood mononuclear cell
PFA: Paraformaldehyde
RRMS: Relapsing remitting multiple sclerosis
SSC: Side scatter
TLR: Toll-like receptor
VZV: Varicella zoster virus
