# Supplementary material for: The expression profile of virus-recognizing toll-like receptors in natural killer cells of Cypriot multiple sclerosis patients
Source: BMC Res Notes. 2020 Sep 29;13:460. doi: 10.1186/s13104-020-05300-1 (PMC7526110; doi:10.1186/s13104-020-05300-1)
Supplement: Supplementary file 1 — Additional file 1: Table S1. Demographic and clinical characteristics of MS patients and healthy controls. The Mann-Whitney U test was used for age matching, and the Fisher’s exact test was used for gender matching. [file 13104_2020_5300_MOESM1_ESM.docx]

**Table S1:** Demographic and clinical characteristics of MS patients and healthy controls. The Mann-Whitney U test was used for age matching, and the Fisher’s exact test was used for gender matching.

| **Features** | **MS patients (n=19)** |  | **HCs (n=20)** | ***p*-value** |
| --- | --- | --- | --- | --- |
| Age (mean[SD]) | 50.11 ±12.10 |  | 47.1 ±10.38 | 0.534 |
| Gender (male/female) | 8/11 |  | 8/12 | >0.999 |
| Disease course (RR/SP/PP) | 19/0/0 |  | N/A |  |
| Duration of disease (years) [median (interquartile range)] | 14.00 (5.00 – 20.50) |  | N/A |  |
| EDSS [median (interquartile range)] | 2.00 (1.50 – 3.25) |  | N/A |  |
| Type of treatment [n (%)] |  |  | N/A |  |
| IFNβ (IFNβ-1a or IFNβ-1b) | 8 (42.11) |  |  |  |
| Natalizumab | 1 (5.26) |  |  |  |
| Teriflunomide | 1 (5.26) |  |  |  |
| None | 9 (47.37) |  |  |  |
| **MS**: Multiple sclerosis; **HCs**: Healthy Controls; **RR**: Relapsing Remitting MS; **SP**: Secondary Progressive MS; **PP**: Primary Progressive MS; **SD**: Standard Deviation; **N/A**: Not Applicable | | | | |
